# The intricate relationship between gut and kidney

**DOI:** 10.1590/1678-4685-JBN-2018-00020001

**Published:** 2018-06-21

**Authors:** Regiane S. Cunha, Andréa E. M. Stinghen

**Affiliations:** 1 Universidade Federal do Paraná, Departamento de Patologia Básica, Laboratório de Nefrologia Experimental, Curitiba, PR, Brasil.

In chronic kidney disease (CKD), the intestinal epithelium has compromised functions, including its ability to act as a barrier and to contribute to gut homeostasis. Data from the literature have shown that changes in the gut barrier contribute to systemic inflammation in patients with CKD. However, these pathological processes have not yet been fully elucidated. The dysfunction of the intestinal epithelium can be explained, at least in part, by the effects induced by uremia resulting from CKD on intestinal epithelial cells, such as enterocytes and colonocytes^1^^-^ 7.

Considering the crucial role of the intestinal epithelium barrier as well as its intrinsic relationship with the immune system, Andrade *et al*. evaluated the effects of uremic serum on colonocytes in vitro, a study published in the present issue of the Brazilian Journal of Nephrology. For this purpose, the authors incubated colonocytes with the serum of healthy individuals, patients on conservative treatment, and patients on hemodialysis (HD), pre- and post-HD. One of the main findings of this study is the significant increase of proinflammatory cytokine interleukin-6 (IL-6) expression by the colonocytes that were incubated with the serum of both pre- and post-HD patients. Corroborating these findings, Lau *et al*. found an increase in the expression of inflammatory molecules, such as the monocyte chemoattractant protein-1 (MCP-1) and cyclooxygenase-2 (COX-2) proteins, and a greater infiltration of leukocytes in the colon of nephrectomized rats^1^. These data, therefore, demonstrate that the uremic environment is able to modulate the inflammatory response in the gut barrier. In addition, Andrade *et al*. did not observe a significant difference in the production of reactive oxygen species (ROS) and expression of toll-like receptors (TLR), proteins that recognize bacterial components and are important for gut homeostasis^7^.

The intestinal epithelium may present an impairment of the intercellular junctions and consequent increase in permeability under pathological conditions. Andrade *et al*. investigated this process through the evaluation of transepithelial electrical resistance of the colonocytes, but no significant difference was found when the cells were exposed to uremic serum. However, Vaziri *et al*. demonstrated that colonocytes exposed to uremic serum from HD patients presented low transepithelial resistance and a significant reduction in the expression of intercellular junction proteins, such as claudin-1, occludin, and zonula occluden-1 (ZO-1)^2^. In another study conducted by Vaziri *et al*., the same effect was observed when colonocytes were exposed to urea, a uremic toxin that has been extensively studied in recent years^3^. Similarly, Lau *et al*. observed in vivo a reduction of claudin-2 expression in colonic tissue of nephrectomized rats^1^. Together, these studies strongly suggest that uremia affects negatively the integrity of the gut barrier.

Gut barrier dysfunction allows bacterial translocation, which is the entry of bacteria and products derived from the microbiota, such as endotoxins, to the bloodstream. In fact, clinical studies have demonstrated increased bacterial translocation that is also associated with inflammation in patients with CKD^4^. Moreover, endotoxins, in particular, are associated with hypervolemia in patients with CKD and experimental data have also shown that endotoxins can lead to the activation of the immune system^5^^,^ 6.

Regarding intestinal homeostasis, the gut microbiota should be considered since it is associated with epithelial cells and is responsible for the formation of various uremic toxins and their precursors. Exemplifying this multifaceted panorama, we can city the uremic toxins *p*-cresyl sulfate and indoxyl sulfate, closely associated with inflammation and cardiovascular diseases in patients with CKD. In addition, clinical studies have shown that the composition of the microbiota is altered in patients with CKD, with a greater abundance of groups capable of producing uremic toxins. Thus, in recent years, several studies with therapeutic focus have been developed in order to modulate the gut microbiota of patients through prebiotics, postbiotics, and symbiotics, but are still inconclusive and further investigation is necessary^4^^,^ 7.

Based on the study conducted by Andrade *et al*. and in the literature, it seems reasonable to suggest that uremia affects the intestinal epithelium by inducing the inflammatory process and compromising the intercellular junctions.^8^ However, little is known about intestinal epithelial dysfunction and CKD. Recently, the research group of Andrade has published a review article highlighting the need to better understand the relationship between intestinal epithelium, microbiota, and CKD^7^. Therefore, further studies are needed to elucidate the complex cellular and molecular mechanisms modulated by uremia in the intestinal epithelium. Finally, findings in this area may contribute to the development of new therapeutic targets in order to improve patient survival.
